# AgroNova: An Autonomous IoT Platform for Greenhouse Climate Control

**DOI:** 10.3390/s26061861

**Published:** 2026-03-15

**Authors:** Borislav Toskov, Asya Toskova

**Affiliations:** Faculty of Mathematics and Informatics, Plovdiv University, 4000 Plovdiv, Bulgaria; toskov@uni-plovdiv.bg

**Keywords:** Internet of Things (IoT), greenhouse climate control, edge computing, rule-based control, hybrid IoT architecture, context-aware decision support, large language models (LLMs)

## Abstract

This study presents AgroNova—a hybrid IoT architecture for autonomous monitoring and management of microclimate in greenhouse environments. The system combines a capillary wireless sensor network, gateway-level rule-based agents, a server agent, cloud services and an advisory component based on a large language model (LLM) that supports local decision-making by incorporating external contextual information from meteorological services. The proposed architecture was validated through a seven-month deployment in an unheated tomato greenhouse, during which more than 380,000 environmental measurements were collected from five sensor nodes. The system operated continuously under real agricultural conditions, including during temporary internet connectivity interruptions, due to the autonomous gateway-level control and deterministic fallback mechanisms. The analysis of the collected data includes 3110 environmental threshold exceedance events, in which recovery dynamics, reaction latency, and actuator activation frequency were evaluated. The results show that the architecture supports stable autonomous operation under limited actuation conditions, with an average local reaction latency of less than 1 s, while physical actuator operations occur in approximately 2.3% of all control decisions. This behavior reflects a conservative control strategy that limits unnecessary mechanical operations and contributes to stable system operation. The experimental integration of a consultative LLM module within the server-side agent demonstrates the potential for context-enriched decision support using external meteorological data, while final control decisions remain under the authority of the gateway-based deterministic control mechanism. The main contribution of this study is the demonstration of a hybrid IoT architecture that combines edge-level autonomy with context-assisted reasoning, validated through deployment in a real greenhouse environment.

## 1. Introduction

The increasing adoption of Internet of Things (IoT) technologies in agriculture has led to the deployment of distributed sensor systems for monitoring environmental parameters in greenhouses and open-field environments. Most existing IoT solutions rely on centralized cloud-based processing, where data are transmitted to remote servers for analysis and decision-making. This model assumes continuous and reliable internet connectivity, which is often not guaranteed in real agricultural conditions.

Edge computing has emerged as an important alternative, moving part of the intelligent logic closer to the physical devices. This approach reduces latency, limits communication dependencies, and allows systems to continue operating autonomously during temporary connectivity disruptions.

The AgroNova system is built on an agent-based IoT gateway architecture previously developed by the authors [1,2]. This architecture introduces a capillary wireless sensor network model combined with a multi-agent system deployed on an intelligent gateway. In this model, each software agent is responsible for a specific end device and implements local control logic based on predefined rules (rule-based control). Deploying agents on the gateway device enables intelligent decisions to be executed at the edge level and ensures continuous system operation even during temporary internet connectivity loss.

Communication between system components is implemented through an MQTT-based publish–subscribe model, enabling asynchronous message exchange between sensors, agents, and server-side services. This approach facilitates the integration of heterogeneous devices and network technologies while minimizing computational requirements for end devices.

Although gateway-level rule-based agents provide reliable and deterministic control under predefined conditions, they lack higher-level contextual reasoning capabilities. In real greenhouse environments, decisions often depend not only on current sensor readings but also on external factors such as weather forecasts or combined environmental conditions.

In this study, we extend the edge-oriented architecture by introducing a hybrid decision-support layer that integrates:

- a server-side agent;
- a large language model;
- external weather API services.

It is important to emphasize that the consultative LLM module does not replace the deterministic local logic. Instead, it provides contextual recommendations that are validated and executed by the gateway agents within predefined safety mechanisms. The present work has two main objectives. First, it presents the first long-term real-world deployment of the proposed gateway-based multi-agent IoT architecture in an unheated greenhouse. Second, it investigates the feasibility of a conservative integration of LLM-based contextual reasoning within the existing edge system.

The purpose of this study is not to establish comparative superiority over alternative control strategies but to analyze the operational behavior of the proposed architecture under real deployment conditions. The focus is therefore on the architectural integration of autonomous edge agents, consultative server-side reasoning, and real-world deployment in an agricultural environment. This combination enables reliable system operation under limited connectivity conditions while extending the capabilities of deterministic edge controllers through contextual information obtained from external sources.

The main contributions of this study are as follows:

- Edge-centric autonomous control architecture
  The AgroNova platform introduces an edge-centric control architecture where environmental decisions are executed directly at the gateway level instead of relying on centralized cloud services. This design reduces communication latency and enables autonomous system operation even when external connectivity is temporarily unavailable.
- Hybrid control: rule-based reasoning combined with consultative LLM reasoning
  The proposed system combines deterministic rule-based control with an optional consultative reasoning layer based on a large language model. This hybrid approach enables the system to maintain predictable local control while incorporating additional contextual information when needed and available.
- Safe Action Mechanism (LLM module cannot execute unsafe commands)
  The architecture uses a bounded action mechanism that restricts LLM recommendations to a predefined set of admissible actuator commands. This constraint ensures all control actions remain within safe operational limits and prevents the execution of unsafe or undefined commands.
- Continuous autonomous operation under limited connectivity
  The system is designed to operate continuously under intermittent or limited network connectivity. The deterministic mechanism ensures that the gateway-level controller maintains full operational autonomy when external services are unavailable.
- Long-term deployment in real greenhouse environments
  The proposed architecture was evaluated over seven months in an operational, unheated greenhouse. This extended deployment provided empirical observations of system behavior under real agricultural conditions.

## 2. Related Work

### 2.1. Greenhouse Automation and Intelligent Agricultural Systems

Greenhouse automation has been developing rapidly with the integration of Internet of Things (IoT) technologies and intelligent monitoring systems. Wireless sensor networks and distributed sensing infrastructures enable continuous monitoring of environmental parameters and remote management of agricultural facilities [3,4,5]. The development of energy-efficient sensor nodes and communication technologies, such as LoRaWAN, further facilitates the deployment of these systems in agricultural environments [6]. Some authors also examine technological advances in greenhouse design, monitoring infrastructures, and sustainable management strategies [7].

More recent studies integrate artificial intelligence methods into agricultural environments for prediction, optimization, and anomaly detection [8,9]. The concept of data-driven agriculture emphasizes the role of IoT platforms and analytical tools in improving transparency and supporting decision-making processes [10]. However, many of these systems remain highly dependent on cloud services and focus primarily on data collection and analysis rather than autonomous decision making at the edge level.

### 2.2. HVAC and MPC-Based Microclimate Control in Greenhouses

A significant portion of the scientific literature focuses on greenhouse microclimate control using classical and predictive control strategies. Model Predictive Control (MPC), PID-based methods, and optimization algorithms are commonly applied to maintain strict environmental setpoints and improve energy efficiency [11,12]. These systems typically assume the availability of active actuators such as heating installations, cooling systems, forced ventilation, humidification/dehumidification systems, or energy screens.

Energy-efficient and net-zero greenhouse concepts have also been investigated, including systems with thermal energy storage and integration of renewable energy sources [13]. In these configurations, controllers have direct influence over environmental dynamics, and system performance is evaluated by minimizing deviations from setpoints, optimal tracking performance, or reducing energy consumption.

In contrast, the deployment of AgroNova was carried out in a greenhouse without active heating or cooling systems. The actuation space is physically limited to the control of shutters and ventilation openings. Consequently, the objective of the system is not strict environmental regulation through optimal control but rather limited stabilization and autonomous decision-making under seasonal influences and physical constraints. For this reason, MPC- and HVAC-based systems represent a different operational class and cannot be considered directly comparable benchmarks for architectures focused on resilience and operation under limited actuation.

### 2.3. Edge-Based Autonomous IoT Architectures

In addition to centralized control approaches, increasing attention is being given to decentralized IoT architectures capable of operating under intermittent connectivity conditions. Some studies focus on layered communication models, distributed device management, and scalable infrastructure design [4,14]. Similar architectural approaches have been explored in other cyber-physical system domains. For example, the ADEPT (Adaptive Data Environment Processing Toolkit) framework provides dynamic orchestration of data streams in IoT environments for ambient assisted living by optimizing data processing and routing across distributed components [15].

Unlike these systems, which primarily focus on adaptive management of data and services, AgroNova addresses the problem of autonomous decision making and control of physical actuators in agricultural environments under limited connectivity conditions.

In the domain of smart agriculture, edge computing is increasingly recognized as a key factor for reducing latency and enabling local autonomy [16,17,18]. Edge intelligence allows decision logic to be deployed directly on gateway devices, reducing dependence on cloud services and ensuring continued operation during connectivity disruptions [16,18]. Multi-agent system approaches further support distributed orchestration and device-level responsibility within IoT infrastructures [19].

In previous work by the authors [1,2], an intelligent IoT gateway framework was introduced, combining a capillary wireless sensor network with gateway-based multi-agent orchestration and MQTT communication. The architecture enables decentralized decision execution and resilient fallback behavior during internet connectivity interruptions. AgroNova builds upon this validated framework, inheriting the gateway autonomy and deterministic rule-based logic while extending it with an additional reasoning layer.

### 2.4. LLM as a Decision-Support Layer in Cyber-Physical Systems

Recent advances in foundation models and large language models have expanded the application of artificial intelligence to contextual reasoning and the integration of heterogeneous knowledge sources [20,21]. Prompt-based strategies further enhance the adaptability of LLMs in structured decision-making environments [22].

However, the direct use of LLMs as autonomous controllers in cyber-physical systems raises concerns about safety and reliability. Generative outputs must be constrained within a predefined action space and validated through deterministic mechanisms to prevent unsafe physical actions.

In IoT environments, LLMs are therefore increasingly used as consultative decision-support components rather than primary controllers. AgroNova adopts this conservative hybrid strategy—the LLM provides context-aware recommendations based on external weather data, while final actuation decisions remain under the deterministic control of the gateway and its safety constraints. In this way, LLM reasoning functions as a contextual extension of deterministic edge autonomy rather than a replacement for it.

## 3. Materials and Methods

### 3.1. Deployment Environment

The system was deployed in a tomato greenhouse at the Maritsa Vegetable Crops Research Institute in Plovdiv, Bulgaria. The greenhouse does not have active heating, cooling, humidification, or dehumidification systems. Therefore, microclimate regulation is limited to passive ventilation using controllable ventilation shutters.

This physical setup defines a limited actuation space, where the system can influence microclimate dynamics only through ventilation control. As a result, stabilization of environmental parameters depends largely on external climatic conditions and does not permit strict maintenance of predefined setpoints, which is typical for greenhouses equipped with heating, ventilation, and air conditioning (HVAC) systems.

**Figure 1:**
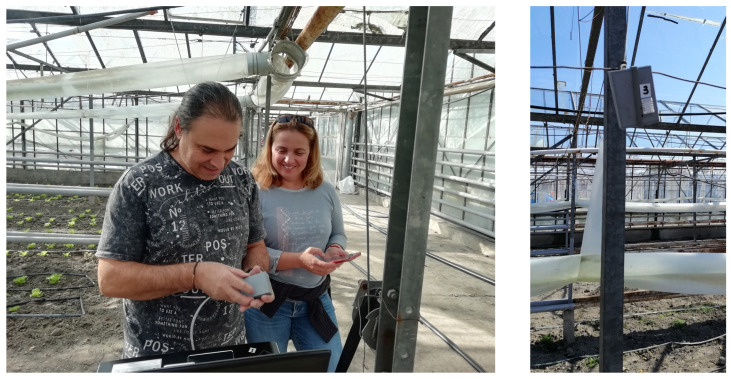
Sensor node installation in greenhouse.

The deployment lasted seven months and generated approximately 380,000 environmental measurements. The physical deployment environment and the installation of the sensor nodes are shown in Figure 1.

**Figure 2:**
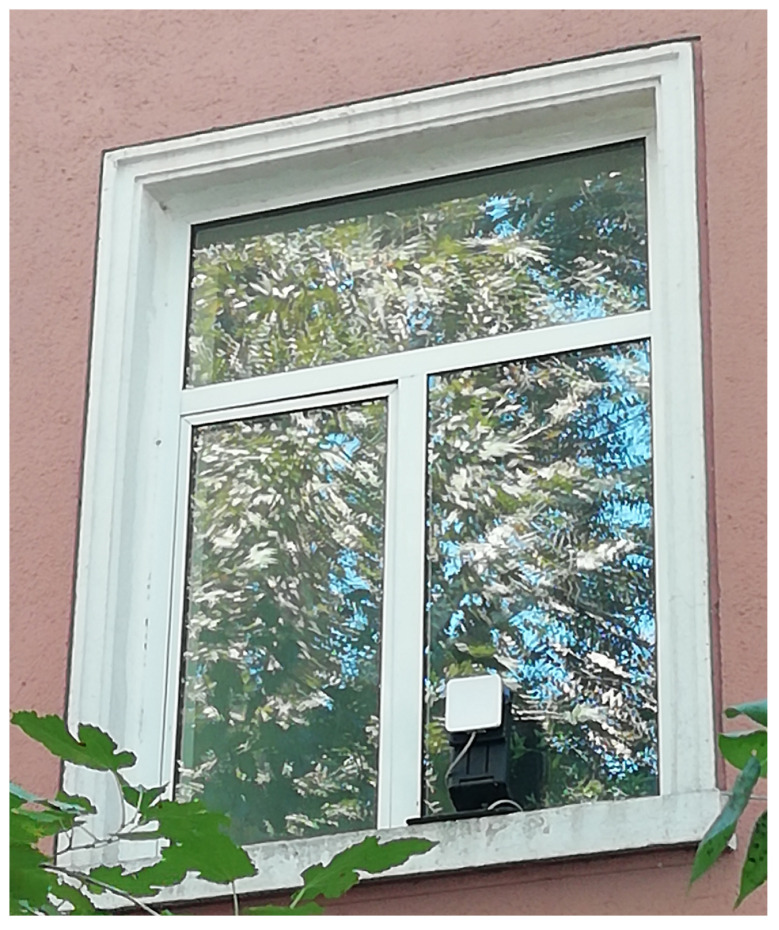
Long-range Wi-Fi antenna setup.

Due to the greenhouse’s location and the absence of direct wired network infrastructure, connectivity was established using a long-range Wi-Fi antenna (Figure 2). This setup introduced realistic variability in network connectivity, further emphasizing the need for gateway-level autonomous operation and deterministic fallback logic. Such an experimental setup allows the system to be evaluated under realistic agronomic constraints and partially unstable network connectivity.

### 3.2. System Architecture Overview

**Figure 3:**
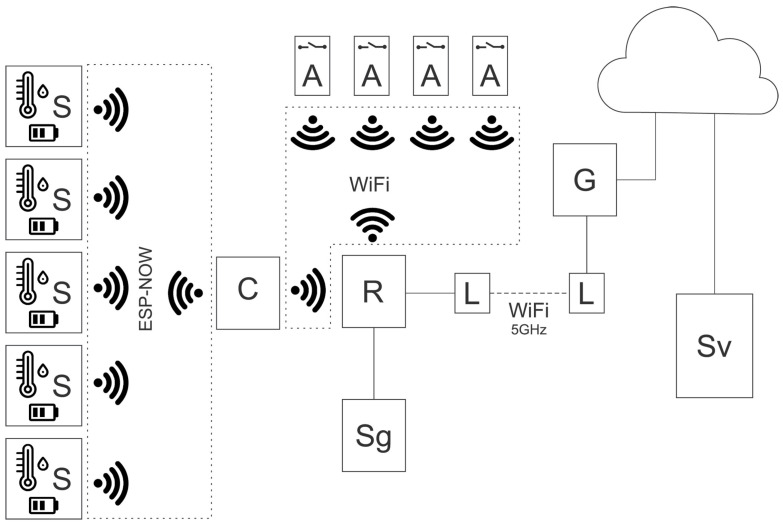
Conceptual architecture of the AgroNova platform (S—sensor nodes; A—actuator; C—coordinator; R—router; G—gateway; L-L—long-range Wi-Fi link; Sv—server; Sg—intelligent gateway).

The overall architecture of AgroNova is shown in Figure 3. The platform uses a multi-layer distributed architecture consisting of a data acquisition layer, a coordination layer, a gateway-level control layer, and a remote cloud service layer.

The main architectural components are:

S—low-power sensor nodes based on ESP32 microcontrollers (Espressif Systems, Shanghai, China), each equipped with a DHT22 digital sensor (Aosong, Guangzhou, China) for measuring temperature and relative humidity;

C—an ESP32-based coordinator that aggregates sensor data via the ESP-NOW protocol;

A—actuator modules based on ESP8266 microcontrollers (Espressif Systems, Shanghai, China), each equipped with two relays to control the electric ventilation shutters;

R—a Wi-Fi router providing local wireless network connectivity;

L—a pair of 5 GHz MikroTik antennas (MikroTik, Riga, Latvia) enabling long-distance wireless communication (up to 12 km) between the gateway and the remote server;

Sg—an intelligent edge gateway (Orange Pi Zero LTS single board computer, Shenzhen Xunlong Software CO., Limited, Shenzhen, China) hosting rule-based agents, MQTT services, and local decision-making logic;

G—an internet gateway providing connectivity to the remote server;

Sv—a remote server hosting a PostgreSQL database (v16 LTS), Grafana visualization tools (v10.4.0), a server-side agent, and a large language model (Mistral-7B-Instruct v0.2, Q4_0 quantization), used for contextual enrichment of decision-making through integration of external information sources.

Sensor nodes (S) periodically transmit environmental measurements to the coordinator (C) using the ESP-NOW protocol. The coordinator forwards aggregated measurements to the intelligent gateway (Sg) via the local wireless network. The gateway executes deterministic rule-based control logic and manages the actuator modules (A).

The long-range Wi-Fi link (L–L) and the internet gateway (G) provide connectivity to the remote server (Sv), which offers data storage, visualization services, and a server-side agent with a consultative LLM module.

All measurements and decision-related events are recorded locally and synchronized with the remote server when network connectivity is available. The intelligent gateway (Sg) serves as the primary decision-making component of the system. Remote cloud services and the consultative LLM module play a supporting consultative role and are used only when necessary and when network connectivity permits.

The software stack of the AgroNova platform includes the following main components:

- Python-based agents (v3.10 LTS) responsible for decision-making at both the local (gateway) and server levels;
- an MQTT broker (Mosquitto v2.0.18) enabling lightweight message exchange between system components;
- a PostgreSQL database (v16 LTS) for storing sensor data and system events;
- Grafana dashboards (v10.4.0) for data visualization and system monitoring;
- a Mistral-7B-Instruct (v0.2) large language model (approximately 7B parameters) with LLaMA-type architecture, running with Q4_0 quantization and a context window of 32k tokens.
- the OpenWeatherMap API (v2.5) used to retrieve external meteorological data;
- ESP-NOW protocol (Espressif Systems Arduino Core, v3.0.7 for ESP32 and v3.1.2 for ESP8266) and Wi-Fi (IEEE 802.11 [23]) protocol enabling wireless communication between sensor nodes, the coordinator, and gateway components of the system.

### 3.3. Local Control Logic

At the gateway level, the architecture supports deploying multiple rule-based agents responsible for monitoring and controlling different environmental parameters. In principle, individual agents can manage specific aspects of the greenhouse environment, such as air temperature, humidity, soil moisture, or nutrient levels.

In the current implementation, a single gateway-level rule-based agent is deployed to monitor air temperature and relative humidity and control the ventilation shutters. This configuration reflects the available sensor infrastructure in the experimental greenhouse.

The main control mechanism is implemented at the gateway level and operates independently of internet connectivity. The local control logic is based on agronomically justified thresholds:

- 15 °C < T < 30 °C
- 60% < RH < 85%.

These thresholds represent conservative upper limits for environmental conditions and were determined in consultation with specialists from the Maritsa Vegetable Crops Research Institute. They were chosen to ensure stable operation within the physical constraints of the experimental environment.

The local control mechanism operates as follows:

- If T > 30 °C or RH > 85%, the ventilation shutters are opened.
- If environmental parameters return to the acceptable range, ventilation is stopped.
- If T < 15 °C or RH < 60%, the system keeps the shutters closed due to the absence of heating or humidification mechanisms.
- If internet connectivity is unavailable, all decisions are made locally based on these rules.

The use of fixed agronomic thresholds is a deliberate architectural decision to ensure stability and reliability in a real agricultural environment. This logic provides:

- predictable system behavior
- control within the physical constraints of the environment
- low reaction latency
- operational independence from cloud services.

### 3.4. Server-Side Control Logic

The remote server hosts a server-side agent that receives MQTT requests from the local gateway agent. These requests include the current greenhouse state (temperature, humidity, ventilation shutter state, and timestamp) along with geographic coordinates. The server-side agent retrieves external weather information for the provided location using the OpenWeatherMap API service. The enriched context, which combines internal greenhouse measurements and external weather conditions, is then provided to a consultative reasoning module as a structured input prompt.

The consultative LLM module uses a locally deployed LLM executed through the Ollama runtime environment on a Linux server. The model used in the experiments is Mistral-7B-Instruct, a transformer-based model with approximately 7 billion parameters and LLaMA-type architecture. The model was executed in a Q4_0 quantized configuration with a context window of 32k tokens.

The gateway agent sends consultation requests to the local Ollama service via HTTP API calls, providing current environmental measurements and external weather information as contextual input.

The prompt template includes the current greenhouse parameters (temperature, humidity, and shutter state) along with external weather information (rainfall and wind conditions) and requests a recommendation limited to predefined actuator actions: open shutters, close shutters, or no action.

The LLM response is constrained to a structured output format that includes environmental context flags and a bounded actuator recommendation. The response contains the following fields:

- is_raining—Boolean value indicating whether rainfall is detected from external weather data
- is_windy—Boolean value indicating strong wind conditions
- action—recommended actuator command, limited to one of the predefined options (open, close, or nothing)
- explanation—a short textual explanation describing the reasoning behind the recommendation

Restricting the response to this predefined schema ensures that the LLM cannot generate arbitrary actuator commands and that all recommendations remain within the admissible control space. The gateway-level rule-based agent parses the structured response and verifies the recommended action before execution. This architectural separation between deterministic local control and consultative LLM reasoning is a safety-oriented design choice.

The inference process is performed locally and does not rely on external cloud APIs.

### 3.5. Decision Switching Mechanism

The hierarchy of decision-making components in the system is as follows:

- Local control by a gateway-level rule-based agent (primary mechanism).
- Recommendations generated by a server-side consultative LLM module (secondary mechanism).

The gateway-level rule-based agent continuously monitors environmental parameters and applies deterministic threshold-based control logic. When the local logic identifies the need for ventilation action and internet connectivity is available, the agent may initiate a consultation request to the server-side agent.

The switching logic operates as follows:

- If internet connectivity is unavailable, decisions are made entirely locally.
- If connectivity is available and an action is required, consultation with the server agent may be initiated.
- If the server response is invalid or delayed, the gateway-level rule-based agent executes its own rule-based decision.

This design ensures continuous operation and control resilience regardless of network conditions. The consultative module extends the contextual awareness of the system without compromising the autonomy of local control.

In this architecture, system intelligence is achieved not through online learning of control policies, but through contextual enrichment of deterministic control using external information sources and consultative reasoning.

### 3.6. Evaluation Metrics

Given the limited actuation space and the absence of active HVAC systems, the evaluation focuses on recovery and stabilization characteristics rather than strict setpoint tracking. The following metrics are defined:

- Threshold exceedance event requiring action—occurs when T > 30 °C or RH > 85%;
- Recovery time—time required for environmental parameters to return below the corresponding thresholds;
- Prolonged deviation—a situation in which threshold exceedance persists longer than a predefined duration;
- Reaction latency—the time between detection of threshold exceedance and activation of the actuator;
- External intervention frequency—the number of events involving consultation with the server-side agent.

These metrics enable a quantitative assessment of autonomous stabilization performance under physical system constraints.

### 3.7. Data Logging and Reproducibility

During deployment, all environmental measurements and actuator states were recorded and stored in the system. Decision-related events were logged with precise timestamps.

The gateway software architecture, decision logic, and action constraints are reproducible in any IoT environment that supports MQTT communication and structured API interaction.

The version of the LLM used, the structure of the prompts, and the definition of the allowable action set are documented to ensure transparency and reproducibility of the consultative decision-making process.

## 4. Results and Discussion

The results in Section 4 provide empirical observations directly related to the main objectives of this study: evaluating an edge-centric hybrid control architecture capable of maintaining autonomous greenhouse operation under real deployment conditions.

### 4.1. Validation in a Real Environment

The AgroNova prototype was deployed for seven months in an unheated greenhouse under real agricultural conditions. The deployment period encompassed diverse climatic conditions, allowing the system to be evaluated under varying environmental parameters.

Each of the five sensor nodes performed measurements approximately every 20 min. In total, 384,052 environmental measurements were collected over 204 days and stored in the PostgreSQL database. This corresponds to approximately 1882 measurements per day, or an average of 76,812 measurements per sensor node.

To illustrate the spatial distribution of the collected dataset, Table 1 summarizes the number of measurements recorded by each sensor node along with the corresponding average environmental values.

The collected data include both daytime and nighttime temperature cycles typical of unheated greenhouses. As a result, the dataset represents long-term continuous monitoring under real agricultural conditions and provides an extensive empirical basis for analyzing system behavior.

The measurements are relatively evenly distributed across the five primary sensor nodes, indicating that the recorded environmental observations are not dominated by a single sensing location and providing spatial coverage of the monitored greenhouse environment.

**Figure 4:**
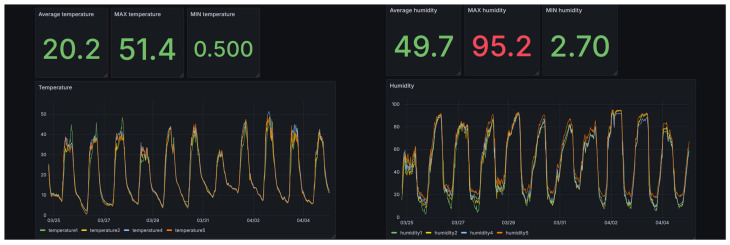
Example daily profiles of temperature and relative humidity recorded by the greenhouse sensor network and visualized in the Grafana monitoring interface.

The system operated continuously throughout the deployment period, except for intentionally induced connectivity interruptions used to validate the autonomous behavior of the gateway. Figure 4 shows typical daily profiles of temperature and relative humidity observed during the deployment period, demonstrating the diurnal environmental variations characteristic of unheated greenhouse environments.

### 4.2. Indicators of Autonomous Operation and Recovery

Despite the absence of active heating or cooling systems, extreme conditions were recorded in only a small fraction of measurements, indicating relatively stable microclimate dynamics throughout most of the deployment period. Table 2 summarizes the statistical distribution of environmental measurements recorded during the deployment period. The distribution of temperature measurements indicates that the greenhouse environment remained within moderate temperature ranges for most of the experiment. Approximately 96.5% of all observations fall within the interval 0–30 °C, while temperatures above 30 °C were recorded in only 3.25% of measurements. Sub-zero temperatures were rare, representing only 0.26% of the dataset. About 36% of observations fall within the agronomically optimal temperature range for greenhouse tomato cultivation (15–30 °C).

For relative humidity, values below 60% were frequently observed. The greenhouse is unheated, does not include a humidification system, and relies on natural ventilation through window openings. Under these conditions, relative humidity may decrease when daytime temperature rises and air exchange occurs with the outside environment. The control system primarily regulates temperature and prevents excessive humidity through ventilation rather than maintaining a specific humidity setpoint.

The results show that only 1.91% of humidity measurements exceeded the 85% threshold, indicating that high-humidity events were relatively infrequent during the deployment period. When such events occur, the ventilation control mechanism can reduce excessive humidity; however, the system cannot actively increase humidity when it is low.

**Figure 5:**
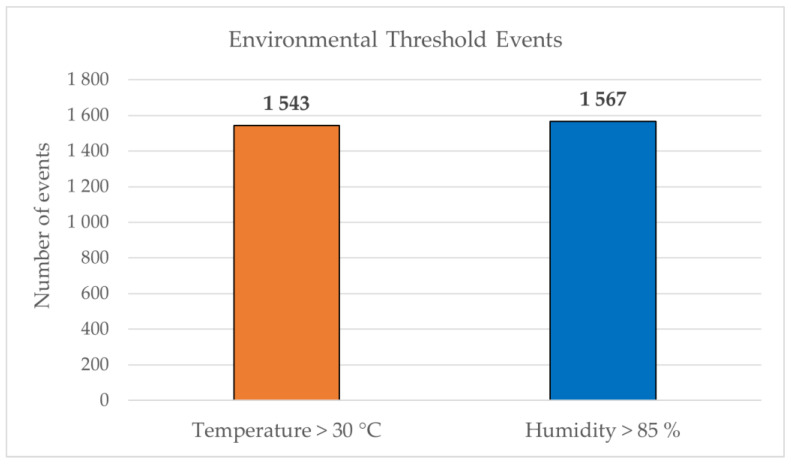
Distribution of temperature (T > 30 °C) and relative humidity (RH > 85%) threshold exceedance events recorded during the seven-month deployment period.

To evaluate stabilization performance under limited actuation, the analysis focuses on exceedance events of the upper environmental thresholds (T > 30 °C and RH > 85%), where mitigation of microclimatic deviations through ventilation is physically possible without active heating or cooling systems. Consequently, the evaluation emphasizes operational stability and recovery behavior rather than strict tracking of predefined setpoints. A total of 3110 environmental threshold exceedance events were observed during the deployment period. These events provided numerous real-world situations in which the automatic control logic was activated. When grouped into continuous exceedance episodes, this corresponds to 1543 temperature events and 1567 humidity exceedance events. As shown in Figure 5, temperature and humidity threshold exceedance events occurred with similar frequency during the monitoring period. On average, approximately 7–8 threshold exceedance events were recorded per day.

For each exceedance episode, the following metrics were calculated (Table 3):

- Latency between event detection and action execution
- Recovery duration (time required for environmental parameters to return below the threshold)
- Frequency of deviations per day.

The average reaction latency for locally executed actions is below one second. LLM-assisted decisions introduce additional latency of 6 to 26 s, caused by network transmission and API request processing.

The average recovery time after exceeding the upper threshold is 7.57 h. It should be noted that the recovery duration reflects the natural dynamics of passive ventilation under the influence of external climatic factors, rather than active microclimate regulation. In the absence of heating, ventilation, and air-conditioning (HVAC) systems, stabilization remains constrained by external temperature and humidity conditions.

Therefore, recovery metrics describe limited mitigation dynamics rather than strict setpoint tracking.

During the deployment period, the gateway-level agent recorded 5180 control decisions in the local execution log (Table 4). On average, the system evaluated environmental conditions about 25 times per day. Of all decisions, 119 resulted in physical actuator operations, corresponding to commands to open or close the ventilation shutters. Most evaluations (97.7%) resulted in a “no action” decision, indicating that environmental conditions remained within acceptable limits for most of the observation period.

The observed ratio between the total number of control decisions and the number of actual actuator operations, approximately 2.3% actuator activation, indicates that the architecture effectively limits unnecessary operations while maintaining sensitivity to significant environmental deviations.

This behavior confirms that the gateway-oriented control architecture maintains a conservative control strategy, in which physical operations are performed only when genuinely required. This reduces mechanical stress on actuators and supports long-term operational stability.

### 4.3. Communication Robustness and Connectivity Behavior

The long-range Wi-Fi infrastructure introduced realistic variability in network stability. During temporary Internet outages, the gateway continued to operate autonomously using deterministic rule-based logic.

No actuator deadlock or system halt occurred during connectivity interruptions. All decisions during offline periods were executed locally and logged for later synchronization.

This confirms the resilience of the gateway-centric control architecture.

### 4.4. Energy Sustainability and Sensor Reliability

The energy sustainability of the sensor nodes was evaluated by analyzing the frequency of device restarts and the maximum number of measurements performed within a continuous operating session. Table 5 summarizes the observed energy performance of the sensor nodes.

Sensor nodes with identifiers 2 and 4 achieved more than 15,000 measurements per session without restart, indicating stable energy efficiency. The remaining nodes recorded a limited number of restarts, including one case caused by an external physical event (flooding resulting from mechanical damage to the greenhouse structure).

These observations indicate that the system maintains stable battery-powered operation and enables planning of preventive maintenance based on observed device behavior.

### 4.5. Evaluation of the LLM Consultation Mechanism

Due to the late integration of the consultative LLM module and its reliance on network connectivity, the number of events involving consultation with the LLM remains limited relative to the overall deployment period. Analysis of the complete system log files indicates that most control decisions were executed locally by the gateway agent. The consultative LLM module was activated only when the gateway agent detected situations requiring contextual interpretation beyond the predefined local rule set.

Over a one-month period of LLM-enabled operation, nine consultation events were recorded in the system logs. In all observed cases, the LLM recommendations remained within the predefined admissible actuator command set and did not produce any unsafe or contradictory actions. The final actuation decisions were verified and executed by the gateway-level rule-based agent. This behavior confirms that the LLM functions solely as a consultative decision-support component rather than as the primary system controller.

The distribution of LLM recommendations shows that approximately 67% of cases resulted in a recommendation to maintain the current ventilation state, while about 33% recommended opening the ventilation shutters. No recommendations to close the shutters were observed.

Due to the limited number of consultation-triggered cases, the available observations are insufficient to support a statistically meaningful classification-style evaluation using metrics such as accuracy or macro-F1. For this reason, the LLM-related results are presented as exploratory operational observations rather than as a benchmark comparison.

All recorded LLM consultation events were manually inspected to verify the consistency of the generated recommendations with the environmental conditions recorded in the system logs and with the predefined agronomic constraints embedded in the control logic. The inspection confirmed that all recommendations remained within the admissible action set and were consistent with the observed environmental context.

Table 6 presents several representative scenarios from the system logs that illustrate the interaction between the gateway-level rule-based agent and the consultative LLM module under various environmental and connectivity conditions. These examples are taken from the recorded consultation events and are intended to illustrate the operational behavior of the hybrid decision mechanism rather than serve as a statistical evaluation.

In one scenario, the LLM recommended keeping the ventilation shutters closed despite the temperature exceeding the threshold because external meteorological data indicated rainfall. This case illustrates the potential for context-sensitive interpretation of conditions that deterministic threshold rules alone cannot capture. The LLM responses also included brief textual explanations describing the reasoning behind the recommendation (e.g., referencing rainfall conditions), which enhances the interpretability of the decision process.

The consultative LLM module can incorporate external contextual information, such as rainfall or wind conditions, when generating recommendations, while the gateway-level rule-based agent relies solely on internal environmental measurements. This mechanism enables the system to extend its contextual awareness without compromising the autonomy and deterministic control implemented at the gateway level.

The system logs further indicate that decision-making based on local rules takes less than one second, while LLM-assisted consultations add a latency of 6 to 26 s due to network transmission and external service processing.

The current LLM-related results should be interpreted as initial empirical observations of the behavior of the hybrid consultative mechanism, rather than as a statistically validated comparative evaluation of decision quality. A more rigorous quantitative evaluation is planned for future research, including replay-based analysis of the complete dataset, expert labeling of consultation scenarios, and controlled comparison among local, hybrid, and weather-informed operation modes.

## 5. Conclusions

This study presents AgroNova, a hybrid IoT architecture for autonomous monitoring and control of greenhouse microclimate. The system integrates a capillary wireless sensor network, gateway-level rule-based agents, a server-side agent, cloud services, and a consultative LLM component, which supports local decision-making by incorporating external contextual information from meteorological services.

Results from a seven-month deployment in an unheated tomato greenhouse demonstrate the operational robustness and practical applicability of the proposed architecture under real agricultural conditions. The system operated continuously throughout the observation period, including during temporary internet connectivity interruptions, due to autonomous gateway-level control and deterministic fallback mechanisms. Over 204 days, more than 380,000 environmental measurements were collected, enabling analysis of threshold exceedance events, recovery dynamics, reaction latency, and actuator activation frequency.

The results show that the proposed architecture supports stable autonomous operation through a conservative control strategy, in which physical actuator operations are performed only when genuinely required. This behavior reflects an architectural approach oriented toward reliability, limited actuation, and system resilience under varying external climatic conditions.

In this study, an experimental extension incorporating a consultative LLM module was also evaluated. This component operates within a constrained action space and provides context-enriched recommendations that incorporate external meteorological data. However, the final control decision remains at the gateway level, ensuring deterministic behavior and safe operation in cases of network unavailability or invalid responses from external services. Due to the late integration of the consultative LLM module, the number of LLM-assisted events is limited, and the results should therefore be interpreted as exploratory.

The main contribution of this work is the demonstration and long-term real-world validation of a hybrid IoT architecture for greenhouse climate control, in which edge-level autonomous control is complemented by an experimentally evaluated consultative reasoning component. The results are based on a study conducted in an unheated tomato greenhouse and primarily demonstrate the operational feasibility of the proposed architecture, rather than its universal applicability across agricultural environments. The findings should be interpreted as observations of architectural behavior under real deployment conditions, not as a controlled comparative evaluation of alternative control strategies.

Future work will include systematic replay-based evaluation using the full dataset, comparative experiments with alternative control strategies, and further investigation of context-assisted decision-making in agricultural cyber-physical systems.

## Figures and Tables

**Table 1:** Distribution of sensor measurements across sensor nodes.

Sensor Node	Measurements	Percentage [%]	Avg. Temperature [°C]	Avg. Humidity [%]
1	78,012	20.71	15.55	35.93
2	78,792	20.92	15.66	36.25
3	69,743	18.51	15.76	33.63
4	79,268	21.05	15.81	37.09
5	78,237	20.81	15.16	40.27

**Table 2:** Distribution of environmental measurements during deployment.

Metric	Value
Temperature < 0 °C	0.26%
Temperature within optimal range 15–30 °C	36.0%
Temperature > 30 °C	3.25%
Humidity < 60%	95.1%
Humidity within optimal range 60–85%	2.97%
Humidity > 85%	1.91%
Average temperature events per day	7.28
Average humidity events per day	7.39
Temperature threshold events	1 543
Humidity threshold events	1 567

**Table 3:** Control response and exceedance recovery metrics.

Metric	Value
Local reaction latency	<1 s
LLM-assisted decision latency	6–26 s
Mean recovery time	7.57 h
Average threshold exceedance events per day	7.3

**Table 4:** Autonomous control activity.

Metric	Value
Total control decisions	5180
Physical actuator actions	119 (2.3%)
Open shutter commands	60
Close shutter commands	59
No action decisions	5061 (97.7%)
Average control decisions/day	25.4
Average actuator actions/day	0.58 (2.3%)

**Table 5:** Sensor node battery lifetime.

Sensor ID	Number of Restarts (Battery Empty)	Max. Measurements per Session
1	2	8149
2	0	15,209
3	2	6605
4	0	15,220
5	1	11,656

**Table 6:** Representative scenarios illustrating interaction between the gateway-level rule-based agent and the consultative LLM module under different environmental and connectivity conditions.

T [°C]	H [%]	Shutter State	Connection	Rain	Wind	Action	Decision Source
24.8	71.0	Closed	No	–	–	No action	Gateway-level rule-based agent
30.1	43.0	Closed	Yes	No	No	Open shutters	Consultative LLM module
31.0	43.0	Opened	Yes	No	No	No action	Consultative LLM module
20.1	76.3	Opened	No	–	–	No action	Gateway-level rule-based agent
13.2	53.1	Opened	No	–	–	Close shutters	Gateway-level rule-based agent
31.1	65.5	Closed	Yes	Yes	No	No action	Consultative LLM module

## Data Availability

The data presented in this study are not publicly available due to confidentiality and operational restrictions but are available from the corresponding author upon reasonable request.
